# A meta-analytical assessment of *STK39* three well-defined polymorphisms in susceptibility to hypertension

**DOI:** 10.1038/srep25290

**Published:** 2016-05-04

**Authors:** Hualing Yang, Lingyang Ye, Qingxiang Wang, Dongmiao Cai, Qumin Chen, Hongming Pan, Zhanxiang Wang

**Affiliations:** 1 Department of Anesthesiology, The First Affiliated Hospital of Xiamen University, Xiamen, Fujian, China; 2 Basic Medical Science College, Qiqihar Medical University, Qiqihar, Heilongjiang, China; 3 Department of Neurosurgery, The First Affiliated Hospital of Xiamen University, Xiamen, Fujian, China

## Abstract

Serine/threonine kinase 39 gene (*STK39*) is one of the promising hypertension-susceptibility genes identified by a genome-wide association study in 2009, whereas subsequent validation in other ethnic groups is unsatisfactory, with inconsistent and inconclusive findings. We therefore aimed to meta-analytically assess the risk prediction of *STK39* three polymorphisms, rs6749447, rs35929607 and rs3754777, for primary hypertension. Literature search and data collection were independently completed by two authors. Nine articles were pooled in this study. Overall analyses failed to see any significant associations of rs6749447, rs35929607 and rs3754777 with hypertension risk (odds ratio: 1.27, 0.95 and 1.21; P = 0.270, 0.507 and 0.153, respectively), and there was evident heterogeneity for three comparisons (*I*^2^ > 80%). Meta-regression analyses indicated that smoking was a significant risk factor for the association of rs3754777 with hypertension (P = 0.017). As reflected by the Begg’s and Filled funnel plots, as well as Egger’s tests, there were low probabilities of publication bias. In conclusion, our meta-analytical findings suggest that *STK39* might not be a hypertension-susceptibility gene.

Since serine/threonine kinase 39 gene (*STK39*) was first identified as a hypertension-susceptibility gene by a genome-wide association study in 2009^1^, it has become a major target of ongoing studies to unravel which genetic loci in *STK39* can predispose individuals at risk for elevated blood pressure^2^,^3^,^4^. Biologically, *STK39* gene encodes a serine/threnine kinase termed as a Ste20-related proline/alanine-rich kinase (SPAK). Compelling evidence from *in vitro* studies indicates that SPAK binds to and phosphorylates Na^+^-dependent cation chloride cotransporters, which can mediate renal NaCl reabsorption and further control salt transport in osmotic cell volume regulation^5^,^6^,^7^, a critical process implicated in the pathogenesis of hypertension. Human *STK39* is located on chromosome 2q24.3, comprises 18 exons and spans approximate 300 kb^8^. More than ten thousand single nucleotide polymorphisms (SNPs) are identified in *STK39* (http://www.ncbi.nlm.nih.gov/SNP/snp_ref.cgi?locusId=27347). Thereof, three intronic SNPs, rs6749447, rs35929607 and rs3754777, have been extensively evaluated in association with hypertension, yet with inconsistent and inconclusive findings, likely due to the genetic heterogeneity of populations selected, the possible small effect of single SNP on hypertensive risk, the relatively small sample size in individual studies, and the lack of evidence in populations other than Asian and White origins^3^,^9^,^10^. To help clarify this issue, we set out to conduct a systematic meta-analysis to better evaluate the risk prediction for primary hypertension in relation to three aforementioned SNPs in *STK39*, according to the guidelines listed in the PRISMA (preferred reporting items for systematic reviews and meta-analyses) statement (for more information, please visit the website http://www.prisma-statement.org/).

## Results

**Figure 1:**
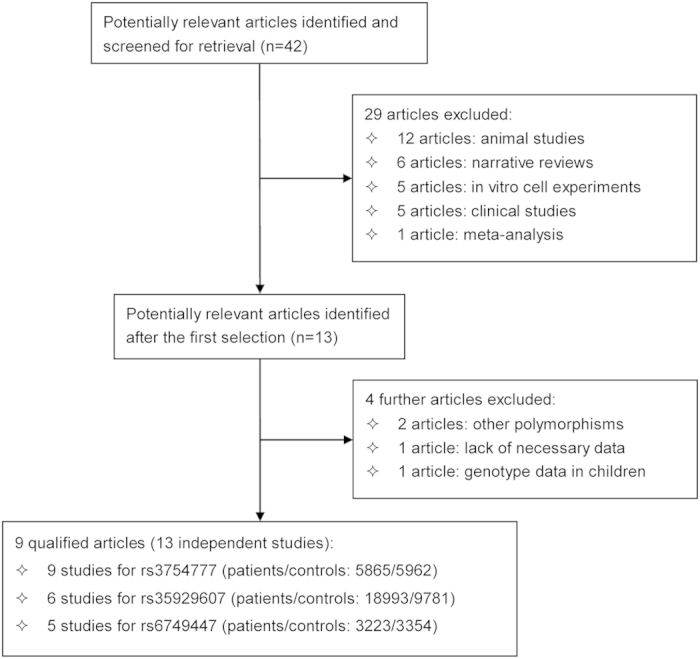
The PRISMA flow chart with the selection of qualified articles.

Out of 42 potentially eligible articles identified from the extensive literature search, 9 articles that satisfied the pre-set inclusion criteria were pooled in this meta-analysis^3^,^4^,^8^,^9^,^10^,^11^,^12^,^13^,^14^. The PRISMA flow chart detailing the selection of qualified articles is shown in Fig. 1. Eight of 9 qualified articles were published in English language, and one in Chinese language^11^. Two articles that included 4 and 2 independent studies were analyzed separately^4^,^8^. Therefore, there were 13 qualified independent studies in the final analysis, including 5, 6 and 9 studies for rs6749447 (patients/controls: 3223/3354), rs35929607 (patients/controls: 18993/9781) and rs3754777 (patients/controls: 5865/5962) in association with hypertension, respectively. The average minor allele frequencies of rs6749447, rs35929607 and rs3754777 were 27.02%, 32.32% and 23.58% in patients and 23.18%, 35.89% and 20.92% in controls, respectively. The demographic characteristics and the lipid profiles of all study populations are presented in Tables 1 and 2, respectively. The genotype accounts of three examined SNPs in *STK39* between patients and controls are summarized in Supplementary Table S1.

**Figure 2:**
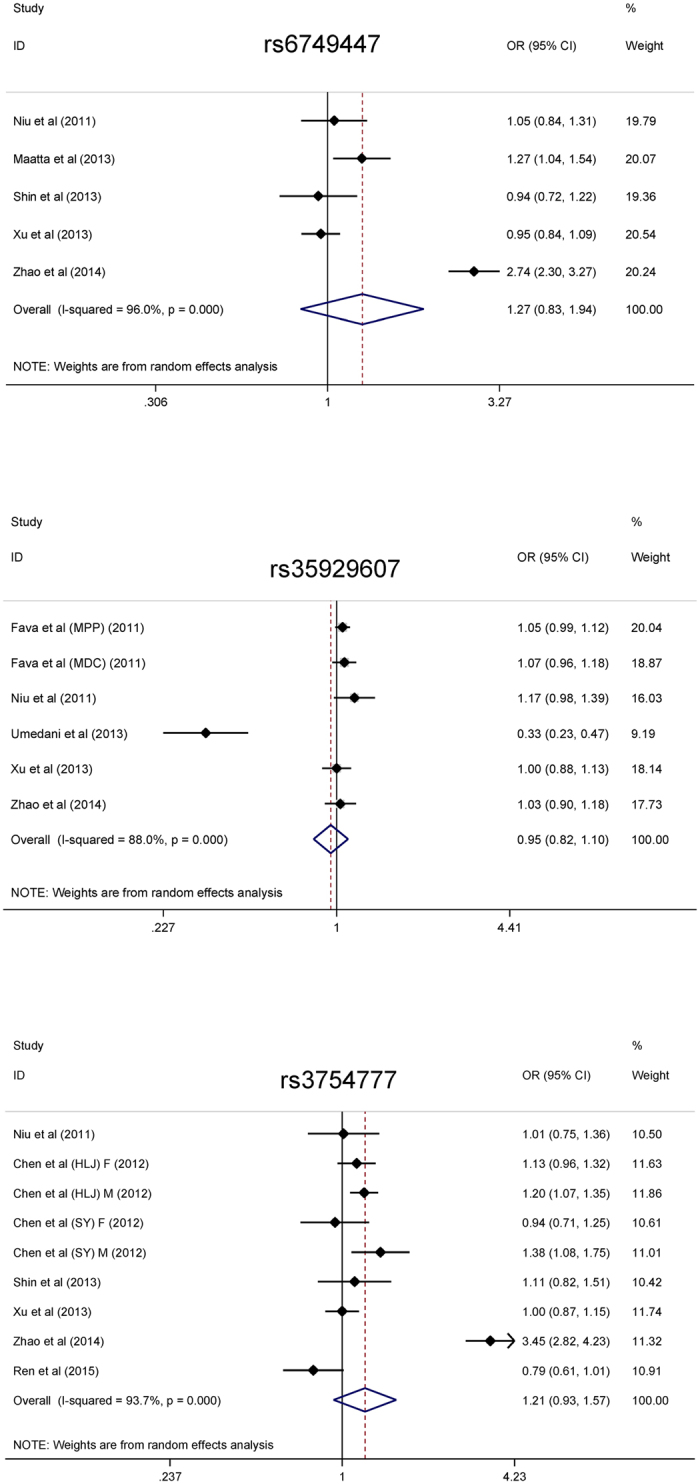
The forest plots of allele comparisons for three examined SNPs.

**Figure 3:**
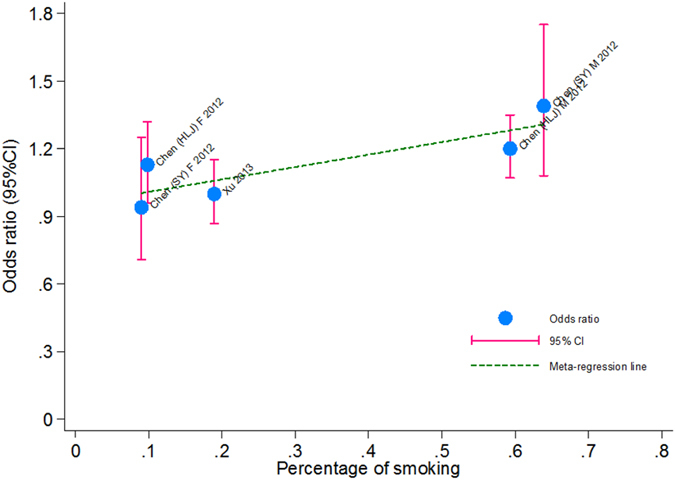
The regression of smoking on individual effect-size estimates of rs3754777 for hypertension risk.

As discrete genotype counts are not usually available in some studies, only alleles were calculated and pooled in this meta-analysis (Fig. 2). Overall, there was no indication of three examined SNPs in susceptibility to hypertension (for rs6749447, rs35929607 and rs3754777: OR = 1.27, 0.95 and 1.21; P = 0.270, 0.507 and 0.153, respectively), and there were significant heterogeneity (*I*^2^ > 80%). In addition, considering the high misclassification rate of restriction fragment length polymorphism (RFLP) method used by two studies^12^,^13^, we conducted a sensitivity analysis by excluding these two studies and there were no changes for the effect-size estimates. To find the potential causes of significant heterogeneity, meta-regression analyses modeling race, study design, control source, age, gender, body mass index, smoking, drinking, fasting glucose, triglyceride, total cholesterol, and high-density lipoprotein cholesterol as independent variables were done. It is of interest to note that smoking was a significant risk factor for the association between rs3754777 and hypertension, as presented in Fig. 3. With the higher percentage of hypertensive smokers, the effect-size estimates of rs3754777 on hypertension risk became more obvious (regression coefficient: 0.34, P = 0.017).

**Figure 4:**
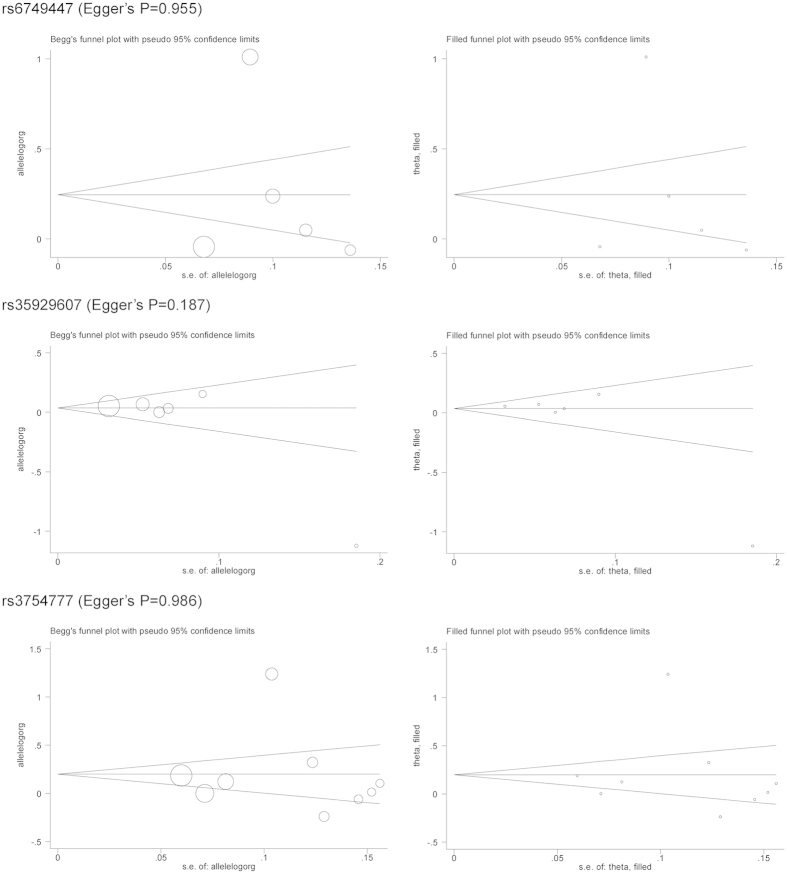
The Begg’s (the left panel) and Filled (the right panel) funnel plots of three examined SNPs.

As shown in the Begg’s and Filled funnel plots (Fig. 4), as well as Egger’s tests, there were low probabilities of publication bias for rs6749447 (P_Egger_ = 0.955), rs35929607 (P_Egger_ = 0.187), and rs3754777 (P_Egger_ = 0.986), and there were no missing studies as indicated by the Filled funnel plots in Fig. 4.

## Discussion

The aim of this meta-analysis was to assess the risk prediction of three promising SNPs, rs6749447, rs35929607 and rs3754777 in *STK39*, for primary hypertension, and none of these SNPs contributed to the significant risk of hypertension. Even though, it is of interest to note that rs3754777 might interact with smoking to make individuals more susceptible to experience hypertension. To our knowledge, this is so far the most comprehensive meta-analysis pivoting *STK39* genetic alterations and hypertension risk from medical literature.

A previous meta-analysis by Xi *et al.* examined only rs3754777 in *STK39* along with its strongly-linked proxies (rs2063958 and rs35929607), and found that rs3754777 was a significant risk factor for primary hypertension in populations of Caucasian and East Asian ancestries^15^. The present updated meta-analysis, however, failed to reproduce this significant finding, as well as for the proxy SNP rs35929607 selected by Xi *et al.*^15^. There are several possible reasons for this divergence, and the first one is that the overall and subgroup estimates in the Xi’s meta-analysis were based on individual effect-sizes of different genetic models of inheritance^15^, which might introduce an analytical bias. To clear this confusion, only allelic model was implemented in the present meta-analysis. Another possibility lies in the incorporation of proxy SNPs in the Xi’s meta-analysis^15^, as the strong linkage disequilibrium in one ethnic group cannot necessarily be extrapolated to that in another ethnic group^16^. For instance in the Xi’s meta-analysis, rs2063958 was indexed as a proxy for rs3754777 (D’ = 0.98)^15^; however as indicated by HaploReg v2 (http://www.broadinstitute.org/mammals/haploreg/haploreg.php), rs2390639 is a more suitable candidate to surrogate rs3754777 as their linkage disequilibrium coefficient D’ reached 0.99. However, the two SNPs, rs2063958 and rs2390639 in the study by Kidambi *et al.*^17^ showed no sign of linkage disequilibrium. A third possibility was that there were duplicated study samples in the Xi’s meta-analysis^15^, such as the MPP study population at baseline and follow-up in the study by Fava *et al.*^4^. To overcome these drawbacks, we separately analyzed the individual contributions of three widely-evaluated SNPs in *STK39* to hypertension, and no observable significance was noticed, suggesting that multiple SNPs in *STK39* mediate blood pressure regulation, with each exerting small effects under a certain environmental condition.

It is worth noting in this study our meta-regression analyses suggested a possible interaction between rs3754777 and smoking in predisposition to hypertension. There is a wide recognition that smoking is an established risk factor for hypertension^18^,^19^. However, a literature search failed to reveal any biological or epidemiological evidence for this interaction. Elucidating the pathophysiological mechanisms underlying rs3754777 and hypertension associated with smoking is beyond the scope of the present study, and it is rational to assume that rs3754777 may influence, under a smoking condition, the functional activation of *STK39* gene, and further control the salt transport.

Several drawbacks for the present meta-analysis need to be acknowledged. First, the sample size in this study is not large enough to ensure adequate statistical power for obtaining a convincing estimate. Second, only hypertension as a binary trait was analyzed and blood pressure as a continuous trait was not due to the insufficient data across qualified studies. Third, the conclusion of this study was merely based on case-controls studies, which might hinder the perhaps cause-effect relationship. Fourth, in spite of no indication of publication bias, there was strong evidence of between-study heterogeneity, and interpreting heterogeneity is still a challenge, as with a majority of meta-analyses^20^. Considering the limited number of studies involved, further subgroup analyses were not conducted, and further large, well-designed studies are required to fully address this issue.

Taken together, we through a comprehensive meta-analysis concluded that *STK39* might not be a hypertension-susceptibility gene. Future studies should incorporate various lifestyle and environmental risk factors such as smoking and drinking in genetic estimation models to precisely define the role of specific genetic variants.

## Methods

### Search strategy

Four databases including Medline (PubMed since 1966), Embase (Excerpta Medica Database since 1980), Wanfang (www.wanfangdata.com.cn/, a Chinese database since 1966) and CNKI (www.cnki.net/, a Chinese database since 1979) were looked through to find studies that were aimed to explore the associations of *STK39* genetic variants with hypertension in human beings on October 25, 2015. The language of qualified articles was confined to English or Chinese. This search process was independently run by two authors (Hualing Yang and Lingyang Ye) on the basis of predefined key terms. Search results were combined with the removal of any duplicate articles.

### Study selection

The title and/or abstract of each article were checked to ensure whether the topic of interest was included, and in case of uncertainty the full text was reviewed according to bespoke selection criteria. Included articles must simultaneously satisfy the following constraints (a) primary hypertension as the clinical endpoint; (b) case-control study design; (c) one or more SNPs of rs6749447, rs35929607 and rs3754777 under investigation; (d) detailed genotype or allele frequencies for examined SNPs between cases and controls. The selection process was completed by two independent authors (Hualing Yang and Lingyang Ye), and any disagreement was resolved through discussion.

### Data abstraction

From each qualified article, relevant data were abstracted by Hualing Yang and subsequently checked by Lingyang Ye, and all data were entered into a predetermined Excel spreadsheet, including the first author’s surname, year of publication, race of study subjects, study design, control source, genotyping method, a list of SNPs under study, genotype or allele counts, sample size, age, male proportion, body mass index, smoking, drinking, fasting glucose, triglyceride, total cholesterol, and high-density lipoprotein cholesterol.

### Statistical analysis

Individual effect-size estimates of *STK39* three SNPs for hypertension risk were pooled in a random-effects model, and odds ratio (OR) and its 95% confidence interval (95% CI) were calculated by the DerSimonian and Laird method^21^. Between-study heterogeneity was measured by the magnitude of *I*^2^ value in terms of percentage, and significant heterogeneity was reported if the *I*^2^ value is greater than 50%, with higher values indicating stronger heterogeneity^22^. Sources of heterogeneity were explored by meta-regression analysis. The probability of publication bias was visually inspected by Begg’s funnel plots and statistically assessed by Egger’s tests at a significance level of 0.10^23^. In addition, the trim-and-fill method was employed to assess publication bias, and this method can be used for estimating and adjusting for the number and outcomes of missing studies in a meta-analysis^24^. The STATA 13.0 software was used to handle all statistical analyses.

## Additional Information

**How to cite this article**: Yang, H. *et al.* A meta-analytical assessment of *STK39* three well-defined polymorphisms in susceptibility to hypertension. *Sci. Rep.* **6**, 25290; doi: 10.1038/srep25290 (2016).

## Supplementary Material

Supplementary Information

## Figures and Tables

**Table 1:** The demographic characteristics of all qualified populations

Author (year)	Race	Design	Source	Genotyping	Sample size		Age (years)		Male		BMI (kg/m^2^)	
					Patients	Controls	Patients	Controls	Patients	Controls	Patients	Controls
Ren *et al.*^11^	Asian	RD	PB	Sequencing	300	300	NR	NR	NR	NR	NR	NR
Zhao *et al.*^9^	Asian	RD	HB	LDR	1009	756	64.48 (8.53)	64.23 (10.13)	0.543	0.538	27.89 (6.29)	23.18 (3.77)
Xu *et al.*^9^	Asian	RD	PB	GenomeLab	1024	1024	57.23 (7.24)	57.01 (7.39)	0.436	0.436	24.65 (3.25)	23.22 (2.88)
Umedani *et al.*^12^	Asian	RD	PB	RFLP	74	454	37.08 (16.42)		0.520		NR	NR
Shin *et al.*^10^	Asian	RD	PB	TaqMan	238	260	50.00 (45–55)	51.00 (45–57)	0.496	0.504	NR	NR
Maatta *et al.*^13^	White	PD	PB	RFLP	447	771	50.00 (0.0)	50.00 (0.0)	0.600	0.570	28.60 (5.1)	25.40 (3.6)
Chen *et al.* (SY) M (2012)	Asian	RD	HB	Sequencing	335	365	56.81 (10.79)	57.01 (12.06)	1.000	1.000	25.01 (3.14)	23.81 (3.18)
Chen *et al.* (SY) F (2012)	Asian	RD	HB	Sequencing	266	244	59.39 (9.60)	56.52 (11.60)	0.000	0.000	24.97 (3.54)	23.39 (3.18)
Chen *et al.* (HLJ) M (2012)	Asian	RD	HB	Sequencing	1330	1641	56.99 (10.76)	57.69 (10.57)	1.000	1.000	25.39 (3.19)	24.20 (3.20)
Chen *et al.* (HLJ) F (2012)	Asian	RD	HB	Sequencing	815	812	61.75 (9.05)	58.52 (8.80)	0.000	0.000	25.14 (3.49)	23.82 (3.38)
Niu *et al.*^14^	Asian	RD	HB	LDR	548	560	NR	NR	NR	NR	NR	NR
Fava *et al.* (MDC) (2011)	White	PD	PB	EPF	3565	2070	57.50 (5.9)		0.417		25.90 (4.0)	
Fava *et al.* (MPP) (2011)	White	PD	PB	EPF	12773	4917	68.20 (5.8)		0.633		27.20 (4.1)	

Notes: RD, retrospective design; PD, prospective design; PB, population-based; HB, hospital-based; LDR, ligase detection reaction; RFLP, restriction fragment length polymorphism; EPF, endpoint fluorescent; BMI, body mass index; M, male; F, female; NR, not reported. Data are expressed either as mean (standard deviation) or median (interquartile range) or percentage.

**Table 2:** The lifestyle and lipid characteristics of all qualified populations.

Author (year)	Smoking		Drinking		Glucose (mmol/L)		TG (mmol/L)		TC (mmol/L)		HDL-C (mmol/L)	
	Patients	Controls	Patients	Controls	Patients	Controls	Patients	Controls	Patients	Controls	Patients	Controls
Ren *et al.*^11^	NR	NR	NR	NR	NR	NR	NR	NR	NR	NR	NR	NR
Zhao *et al.*^9^	NR	NR	NR	NR	6.14 (2.15)	5.33 (1.12)	1.90 (1.04)	1.77 (0.95)	4.59 (1.18)	4.59 (0.91)	1.12 (0.32)	1.24 (0.34)
Xu *et al.*^3^	0.189	0.164	0.229	0.192	NR	NR	2.02 (1.69)	1.64 (1.12)	5.34 (1.01)	5.18 (0.93)	1.41 (0.35)	1.40 (0.32)
Umedani *et al.*^12^	NR	NR	NR	NR	NR	NR	NR	NR	NR	NR	NR	NR
Shin *et al.*^10^	NR	NR	NR	NR	4.72 (4.33–5.22)	4.56 (4.33–4.94)	1.38 (0.96–2.03)	1.16 (0.87–1.78)	5.07 (4.53–5.69)	5.15 (4.53–5.82)	1.22 (0.98–1.50)	1.29 (1.09–1.55)
Maatta *et al.*^13^	NR	NR	NR	NR	NR	NR	1.50 (1.2)	1.20 (0.7)	5.40 (1.0)	5.40 (1.0)	1.60 (0.4)	1.70 (0.4)
Chen *et al.* (SY) M (2012)	0.639	0.514	0.371	0.249	6.37 (2.22)	6.18 (1.86)	NR	NR	NR	NR	NR	NR
Chen *et al.* (SY) F (2012)	0.090	0.107	0.080	0.021	6.61 (2.36)	5.61 (1.62)	NR	NR	NR	NR	NR	NR
Chen *et al.* (HLJ) M (2012)	0.593	0.525	0.473	0.405	6.56 (2.22)	6.27 (2.20)	NR	NR	NR	NR	NR	NR
Chen *et al.* (HLJ) F (2012)	0.098	0.091	0.017	0.023	6.71 (2.39)	6.03 (1.87)	NR	NR	NR	NR	NR	NR
Niu *et al.*^14^.	NR	NR	NR	NR	NR	NR	NR	NR	NR	NR	NR	NR
Fava *et al.* (MDC) (2011)	0.276		NR	NR	NR	NR	NR	NR	NR	NR	NR	NR
Fava *et al.* (MPP) (2011)	0.372		NR	NR	NR	NR	NR	NR	NR	NR	NR	NR

Notes: TG, triglyceride; TC, total cholesterol; HDL-C, high-density lipoprotein cholesterol; M, male; F, female; NR, not reported. Data are expressed either as mean (standard deviation) or median (interquartile range) or percentage.
