# Supplementary material for: A meta-analytical assessment of STK39 three well-defined polymorphisms in susceptibility to hypertension
Source: Sci Rep. 2016 May 4;6:25290. doi: 10.1038/srep25290 (PMC4855189; doi:10.1038/srep25290)
Supplement: Supplementary Information [file srep25290-s1.pdf]

A meta-analytical assessment of *STK39* three well-defined polymorphisms in susceptibility to hypertension

Hualing Yang[1,*], Lingyang Ye[1,*], Qingxiang Wang[1,*], Dongmiao Cai[1], Qumin Chen[1], Hongming Pan[3], Zhanxiang Wang[2,#]

Author affiliations:

[1]Department of Anesthesiology and [2]Department of Neurosurgery, The First Affiliated Hospital of Xiamen University, Xiamen, Fujian, China;

[3]Basic Medical Science College, Qiqihar Medical University, Qiqihar, Heilongjiang, China.

*Shared first authors.

[#]Correspondence should be addressed to:

Zhanxiang Wang, M.D., Ph.D.

Address: Zhenhai Road 55, Siming District, Xiamen 361003, Fujian, China.

Tel & Fax: +86-0592-2139406.

E-mail: 15359252409@163.com.

Short title: *A Meta-Analysis of STK39 and Hypertension*

Disclosure statement: The authors state that they do not have any conflicts of interest.

Supplementary Table S1. The genotype distributions of three examined SNPs in this meta-analysis

Author (year)	rs6749447						rs35929607						rs3754777					
	Patients			Controls			Patients			Controls			Patients			Controls		
	CC	CA	AA	CC	CA	AA	AA	AG	GG	AA	AG	GG	GG	GA	AA	GG	GA	AA
Ren et al (2015)	NR	NR	NR	NR	NR	NR	NR	NR	NR	NR	NR	NR	170	107	23	144	130	26
Zhao et al (2014)	488	435	86	566	175	15	309	499	201	236	377	143	562	384	63	629	119	8
Xu et al (2013)	502	430	92	488	438	98	321	505	198	321	505	198	561	394	69	561	394	69
Umedani et al (2013)	NR	NR	NR	NR	NR	NR	26	44	4	165	11	278	NR	NR	NR	NR	NR	NR
Shin et al (2013)	117	93	28	119	112	29	NR	NR	NR	NR	NR	NR	147	79	12	167	84	10
Maatta et al (2013)	192	204	8	420	324	10	NR	NR	NR	NR	NR	NR	NR	NR	NR	NR	NR	NR
Chen et al (SY) M (2012)	NR	NR	NR	NR	NR	NR	NR	NR	NR	NR	NR	NR	171	138	26	220	127	18
Chen et al (SY) F (2012)	NR	NR	NR	NR	NR	NR	NR	NR	NR	NR	NR	NR	154	97	15	137	92	15
Chen et al (HLJ) M (2012)	NR	NR	NR	NR	NR	NR	NR	NR	NR	NR	NR	NR	703	528	99	953	595	93
Chen et al (HLJ) F (2012)	NR	NR	NR	NR	NR	NR	NR	NR	NR	NR	NR	NR	449	312	54	475	292	45
Niu et al (2011)	386	143	19	394	154	12	227	252	69	256	248	56	460	82	6	469	87	4
Fava et al (MDC) (2011)	1685	1305	233	1987	1203	164	2453	1001	110	1460	549	61	NR	NR	NR	NR	NR	NR
Fava et al (MPP) (2011)	NR	NR	NR	NR	NR	NR	8688	3711	374	3429	1331	157	NR	NR	NR	NR	NR	NR

Notes: NR, not reported.
